# Chemoproteomics Reveals USP5 (Ubiquitin Carboxyl-Terminal Hydrolase 5) as Promising Target of the Marine Polyketide Gracilioether A

**DOI:** 10.3390/md22010041

**Published:** 2024-01-11

**Authors:** Alessandra Capuano, Gilda D’Urso, Michela Aliberti, Dafne Ruggiero, Stefania Terracciano, Carmen Festa, Alessandra Tosco, Maria Giovanna Chini, Gianluigi Lauro, Giuseppe Bifulco, Agostino Casapullo

**Affiliations:** 1 Dipartimento di Farmacia, University of Salerno, Via Giovanni Paolo II 132, 84084 Fisciano, Salerno, Italy; acapuano@unisa.it (A.C.); gidurso@unisa.it (G.D.); mialiberti@unisa.it (M.A.); druggiero@unisa.it (D.R.); sterracciano@unisa.it (S.T.); tosco@unisa.it (A.T.); glauro@unisa.it (G.L.); bifulco@unisa.it (G.B.); 2 PhD Program in Drug Discovery and Development, University of Salerno, 84084 Fisciano, Salerno, Italy; 3 Dipartimento di Farmacia, University of Napoli “Federico II”, Via Domenico Montesano 49, 80131 Napoli, Italy; carmen.festa@unina.it; 4 Dipartimento di Bioscienze e Territorio, University of Molise, Contrada Fonte Lappone, 86090 Isernia, Italy; mariagiovanna.chini@unimol.it

**Keywords:** proteomics, drug affinity target stability, targeted-limited proteolysis, multiple reaction monitoring, marine bioactive compound, molecular docking, ubiquitin carboxyl-terminal hydrolase

## Abstract

Mass spectrometry-based chemical proteomic approaches using limited proteolysis have become a powerful tool for the identification and analysis of the interactions between a small molecule (SM) and its protein target(s). Gracilioether A (GeA) is a polyketide isolated from a marine sponge, for which we aimed to trace the interactome using this strategy. DARTS (Drug Affinity Responsive Target Stability) and t-LiP-MS (targeted-Limited Proteolysis-Mass Spectrometry) represented the main techniques used in this study. DARTS was applied on HeLa cell lysate for the identification of the GeA target proteins, and t-LiP-MS was employed to investigate the protein’s regions involved in the binding with GeA. The results were complemented through the use of binding studies using Surface Plasmon Resonance (SPR) and in silico molecular docking experiments. Ubiquitin carboxyl-terminal hydrolase 5 (USP5) was identified as a promising target of GeA, and the interaction profile of the USP5-GeA complex was explained. USP5 is an enzyme involved in the pathway of protein metabolism through the disassembly of the polyubiquitin chains on degraded proteins into ubiquitin monomers. This activity is connected to different cellular functions concerning the maintenance of chromatin structure and receptors and the degradation of abnormal proteins and cancerogenic progression. On this basis, this structural information opens the way to following studies focused on the definition of the biological potential of Gracilioether A and the rational development of novel USP5 inhibitors based on a new structural skeleton.

## 1. Introduction

The chemical diversity of natural compounds is remarkable and encompasses a wide range of molecules with unique and diversified chemical structures as a result of the evolution and adaptation of living organisms to different environments and conditions. Thus, the study of natural small molecules offers numerous advantages, ranging from drug discovery to the investigation of biological processes and industrial applications. The chemical diversity and potential applications of the natural products make these compounds a valuable resource for researchers, and among them, marine secondary metabolites isolated from algae, corals, sponges, and microorganisms are considered a source of unique chemical entities endowed with potential biological activity. Their wide chemical diversity provides opportunities to discover new drugs, chemicals, and innovative materials. 

Plakortin polyketides represent a structurally and biologically attractive class of marine natural compounds [1,2]. These natural metabolites have been isolated and characterized from various species of marine sponges. In recent years, a series of polycyclic plakortin polyketides, particularly hippolachnin A and gracilioethers, have been identified, attracting significant interest in the scientific community due to their unique molecular structures and promising potential applications.

**Figure 1:**
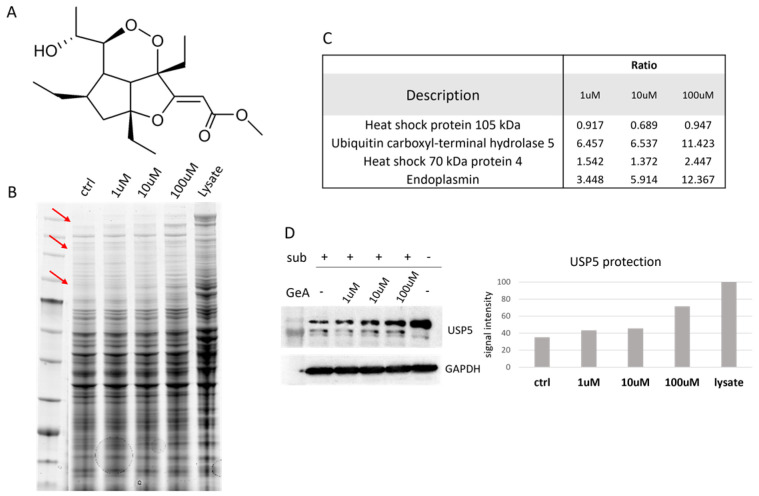
(**A**) GeA molecular structure; (**B**) Coomassie-stained gel with DARTS sample. Red arrows show bands with increasing intensities in accord with increasing amounts of GeA. These bands were supposed to contain GeA putative protein partners. “Ctrl” is the sample treated with subtilisin but without GeA. “Lysate” is the sample untreated with GeA and undigested (see also Figure S4). (**C**) Proteome Discoverer results showing the protection ratio of the four proteins shared among all DARTS experiments at three GeA concentrations. Proteome Discoverer results display the protective ratio of the four proteins consistently observed across all DARTS experiments at different GeA concentrations. (**D**) Western blotting with densitometric analysis. Lysate intensity is set at 100%. (see also Figure S5).

Gracilioether A (GeA, Figure 1) is one of the members of the plakortin family, which was discovered in the deep-sea sponge *Agelas gracilis* [3] and *Plakinastrella mamillaris* [4], along with other related compounds. Gracilioethers A-C exhibited antimalarial activity. 

Given these premises, the present study aimed to carefully investigate the interaction profile of this molecule through proteomic and bio-physical methods to lay the groundwork for the identification of its biological potential or for the development of a new class of USP5 modulators in future studies. We searched for possible protein targets in cancer cell lines with the purpose of exploring the nature of the interaction established between GeA and its biological counterparts.

Our analysis consisted of the application of mass-spectrometry-based chemoproteomic techniques based on limited proteolysis (DARTS and t-LIP-MS), combined with surface plasmon resonance (SPR) and molecular docking studies. The binding of a protein with a small molecule elicits a conformational modification, turning the protein complex into a more stable system that is less susceptible to limited proteolysis compared to the free protein. Drug affinity responsive target stability (DARTS) [5,6] measures this behavior in a limited proteolysis experimental fashion, where the limited digestion, carried out with subtilisin, is used to distinguish the proteins interacting with a small molecule from all the other proteins in a complex mixture as a cell lysate and a following the identification of the potential interacting macromolecule(s). Targeted limited proteolysis coupled to Mass Spectrometry (t-LiP-MS) exploits the same evidence but the addition of second extensive proteolytic digestion with trypsin and the application of Multiple Reaction Monitoring (MRM) mass spectrometry allows the identification of the interacting regions between the protein and the small molecule [7,8,9]. Indeed, the protective effect produced by the small molecule on a particular tryptic peptide residue comprised in the interaction with its target will lead to the identification of these peptides based on their abundance. The application of this combined chemoproteomic approach identified Ubiquitin carboxyl-terminal hydrolase 5 (USP5) as a promising GeA-specific partner. 

Ubiquitination is a significant post-translational modification that regulates a wide range of cellular functions, including cell cycle progression, signal transduction, DNA repair, and many others. The dysregulation of ubiquitination can have severe consequences and is associated with numerous diseases, including cancer [10]. Deubiquitinases (DUBs) [11] are a class of enzymes that play a crucial role in the regulation of ubiquitination by reversing the process of ubiquitin conjugation. While ubiquitination involves the addition of ubiquitin molecules to target proteins, in thedeubiquitination these ubiquitin molecules are removed from the target proteins. The deubiquitination process is essential for the dynamic and precise control of various cellular processes. DUBs are classified into five families, and the Ubiquitin-Specific Proteases (USPs) [12] are the largest ones, covering over 50 members in humans. 

Ubiquitin carboxyl-terminal hydrolase 5 (USP5) is an enzyme that belongs to this family. It is involved in a wide range of cellular processes (stress response, DNA repair, inflammatory response) [13] and has been the subject of extensive research, particularly regarding its association with cancer. In fact, this enzyme is overexpressed in various types of cancer (e.g., breast, prostate), and it seems to act as a tumor promoter due to its ability to stabilize certain oncoproteins. It has been associated with promoting cell proliferation and the Epithelial to Mesenchymal Transition (EMT) in hepatocellular carcinoma, melanoma, and pancreatic ductal carcinoma [13]. The inhibition of USP5’s activity may help to modulate the stability of EMT transcription regulators and, in turn, hinder cancer cell proliferation and EMT, which are crucial steps in cancer progression. Given the important role that this enzyme plays in both tumoral processes and its significant involvement in other pathological processes such as inflammation [14] and neurological [15], it is clear why it is considered an important subject of study. Identifying molecules that interact with USP5 is, therefore, of particular importance to inspire new structures that can be used in therapeutic strategies.

The interaction between GeA and USP5 was validated through a comprehensive approach, encompassing surface plasmon resonance analysis and molecular docking studies. These analyses not only confirmed the specific binding but also provided an in-depth understanding of the interaction between GeA and USP5, further supporting the data obtained through t-LiP-MS.

## 2. Results 

### 2.1. USP5 Identification as Main GeA Protein Partner

As already mentioned, the binding with SM stabilizes the structure of the target protein. This increased stability gives resistance to external perturbations on the protein, such as limited proteolysis under native conditions, allowing it to be identified within a complex system like a cell lysate, where non-specific protein–ligand contacts are minimized due to the high number and variety of proteins in the mixture. 

Thus, the protein target will be less sensitive to the protease in the sample treated with the compound than in the control sample. A first evaluation of the different rates of digestion can be performed on a 1D-SDS-PAGE after protein separation and gel staining. The band of the potential target protein in the samples treated with the SM will result in greater intensity than the control sample due to the higher amount of undigested protein after proteolysis. Proteins can then be identified through the bottom-up proteomic approach.

HeLa cells were mechanically lysed, and the protein extract was incubated with GeA at different concentrations (1 μM, 10 μM or 100 μM) and DMSO as a control sample. Subtilisin, a broad-specificity protease, was added to samples (1:500 *w*/*w* enzyme to proteins ratio, 30 min) to carry out the limited proteolysis step (a sample treated with DMSO and undigested (lysate) was used as a positive control). After quenching the enzyme, proteins were separated through 1D-SDS-PAGE and stained with Coomassie blue (Figure 1B). Gel bands were then excised from the gel and submitted to trypsin digestion for the next bottom-up nano-ESI-UPLC-MS/MS analysis (data available via ProteomeXchange with identifier PXD048098). MS files were examined with the Proteome Discoverer software (version 2.4), providing protein identification and a semi-quantitative analysis that led to the discovery of four GeA potential target(s), three of which belong to the heat shock protein family and the other to the deubiquitinase family (see Figure 1C and Figure S6 and Section 4.3). Among these proteins, USP5 was chosen as the most promising and interesting based on its best proteomic parameters, such as the reported high ratio (protein abundance in the sample treated with GeA/protein abundance in the control experiment) values and the level of protection effect given by GeA even at the lowest concentration (1 μM). These findings were confirmed across three replicates.

These results were then validated by analyzing the samples coming from one DARTS experiment through Western blotting, using a specific antibody anti-USP5. The amount of intact protein remaining after the limited proteolysis step was easily visible from the membrane image and increased with a GeA concentration-dependent trend (Figure 1D, left side). A densitometric analysis of the Western Blots was also carried out to give a more accurate quantitative evaluation of protein resistance to the digestion with subtilisin at the different GeA concentration, using glyceraldehyde 3-phosphate dehydrogenase (i.e., GAPDH) as a loading normalizer [16] (Figure 1D, right side).

### 2.2. Surface Plasmon Resonance of GeA and USP5

DARTS results were confirmed using Surface Plasmon Resonance (SPR) as orthogonal assay. This study allowed to evaluate the interaction kinetics and affinity between ubiquitin-specific proteinase 5 (USP5) protein, immobilized on the SPR chip with GeA as the analyte. The measurement of K_D_ provided insights into the binding strength and can be crucial for understanding the biological significance of this interaction. 

Different concentrations of GeA, spanning from 0 to 100 μM, were injected into the target protein immobilized on the surface of a CM5 chip. 

**Figure 2:**
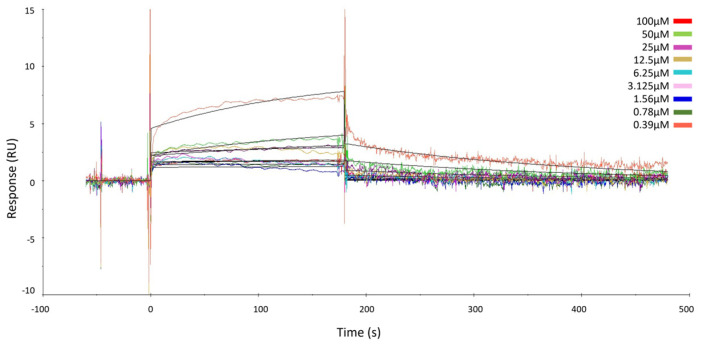
Surface Plasmon Resonance sensorgrams acquired for different concentrations of GeA on immobilized USP5 protein. The response units (RUs) were plotted against time (the abrupt change in signal curvature at 100 µM can be attributed to the absence of significant binding interaction up to 50 µM, while noticeable binding becomes evident at 100 µM).

The results showed that GeA binds the target in a concentration-dependent fashion, just like already proved via the DARTS experiments, and K_D_ was assessed in the micromolar range (K_D_ = 241.00 ± 38.90 µM) (Figure 2 and Figure S8). These data gave evidence of the direct interaction between GeA and USP5 and thus confirmed the results provided by DARTS.

### 2.3. Evaluation of the Interaction Features between GeA and USP5 by t-LiP-MRM-MS

The combination of limited proteolysis (LiP) with the Multiple Reaction Monitoring (MRM) MS scanning mode allowed us to perform a selective analysis of tryptic peptides of the single protein USP5 directly on a complex biological mixture without requiring electrophoresis separation. This procedure provided the analysis and identification of the regions of the protein–ligand complex characterized by higher resistance to proteolysis. As in the DARTS experiments, the LiP-MRM workflow involved the incubation of the SM with a cell lysate and then treatment with subtilisin in limited proteolysis conditions. In the last step, samples were submitted to denaturant conditions and extensive tryptic digestion for the MRM-MS analysis. As a result of this digestion, the mixture will contain fully tryptic peptides (typical peptides produced by trypsin) and half-tryptic peptides, which are species generated by combining proteolytic cleavages from subtilisin and trypsin. The protection of the target protein sites towards the subtilisin cleavages in the samples treated with the SM will improve the amount of the fully tryptic peptides compared to the control sample. Therefore, differences in the relative abundance of fully tryptic peptides will indicate protein structural changes resulting from ligand binding.

Before starting the t-LiP-MRM experiment, a search on the data repository SRMAtlas was needed to obtain information about the comprehensive profile of USP5 fully tryptic peptides and their transitions (MS/MS fragment ions) [17]. Peptides and transitions were then tested and validated on a HeLa tryptic digest, and the final method was built, selecting the best transitions (in terms of intensity and S/N ratio) obtained for each peptide, which were, in turn, marked with the retention time (see Figure S1). 

**Figure 3:**
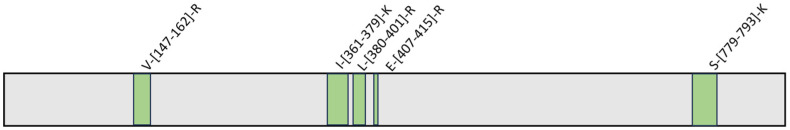
USP5 protected peptides upon binding to GeA identified by t-LiP experiment. (fold change value > 1.5 and *p*-value < 0.05).

Therefore, HeLa lysate samples were incubated with GeA (100 μM final concentration) or DMSO (control sample) and submitted to limited proteolysis in native conditions with subtilisin (an undigested DMSO-treated sample was also taken as a positive control). All samples were then submitted to denaturing conditions for the next extensive tryptic digestion and the desalting step. The resulting mixtures were analyzed via UPLC-MRM-MS on a QTRAP Mass spectrometer. The increasing intensity of five tryptic peptides in the GeA-treated sample compared to the control sample was assessed, revealing the peptide regions protected from subtilisin proteolysis, as reported in Figure 3 (see also Figures S2 and S7 for a complete list of the analyzed peptides). 

### 2.4. Computational Study on the Interactions between GeA and USP5

**Figure 4:**
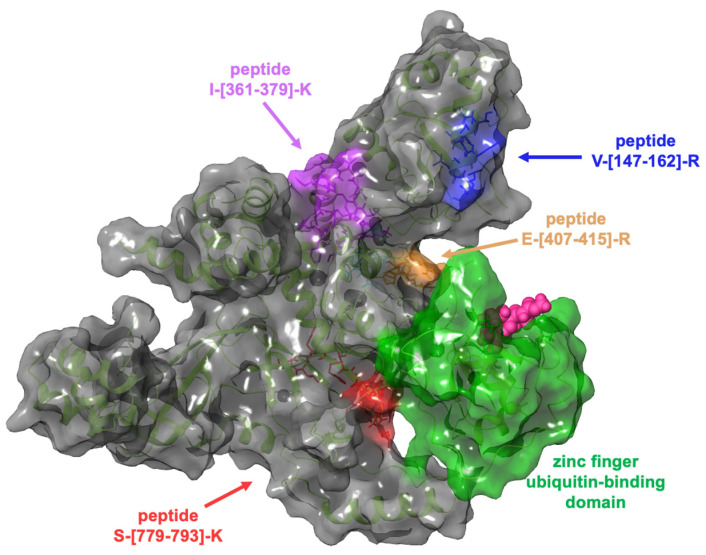
Three-dimensional representation of USP5: the zinc finger ubiquitin-binding domain (coloured in green) is near the tryptic peptide S-[779-793]-K, which was identified in t-LiP-MRM-MS experiments.

Computational studies were performed through molecular docking experiments to rationalize at the molecular level the binding mode of GeA on Ubiquitin carboxyl-terminal hydrolase 5 (USP5). USP5 comprises a zinc finger ubiquitin-binding domain (ZnF-UBD), a functional catalytic domain, two ubiquitin-associated domains (UBAs), and a cryptic zinc finger ubiquitin-binding domain [18]. The t-LiP-MRM-MS experiments highlighted the interaction of GeA with USP5 in a protein region close to the zinc finger ubiquitin-binding domain, as reported in Figure 4.

**Figure 5:**
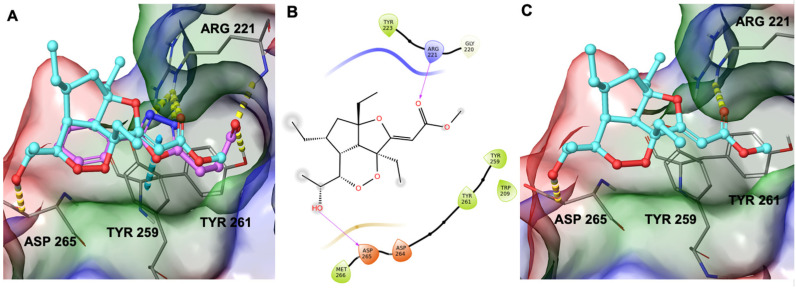
(**A**) Three-dimensional superposition of known inhibitor HHY [18] (coloured by atom type: C violet, O red) with GeA (coloured by atom type: C cyan, O red). (**B**) Two-dimensional representation of GeA binding mode with USP5. (**C**) Binding mode of Gracilioether A in the binding site of the USP5 Zinc Finger Ubiquitin-Binding Domain. Hydrogen bonds are represented as dotted yellow lines while π-stacking interactions are represented as dotted blue lines.

Therefore, during our in silico investigation, we considered that the compound under investigation could bind to ZnF-UBD, which was previously identified as the binding site of new inhibitors of USP5 [19] featuring a carboxylate group. This chemical moiety was responsible for H-bonds with Tyr261 and Arg221 (Figure 5A). Remarkably, the C-terminal diglycine ubiquitin, an endogenous USP5 substrate, establishes the same interaction network. Additionally, these known inhibitors were able to make a π-π stacking interaction with Tyr259 [19]. Given the involvement of the carboxylate group of known inhibitors in the interaction with the protein, we investigated whether the ester group of GeA could establish similar interactions. Starting from these pieces of information, we employed molecular docking experiments on ZnF-UBD (PDB code: 6DXT [19]) using Glide software [20,21,22] (Schrödinger Suite). Computational results showed that GeA can reproduce a similar accommodation of the inhibitor named HHY [19] originally co-crystallized in the protein structure (Figure 5A and Figure S3) and, specifically, it established the key H-bond interaction with Arg221 and an additional H-bond with Asp265 (Figure 5B,C).

In summary, in silico data disclosed that the specific pattern of hydrogen bonds and hydrophobic contacts with the selected binding site represent the driving forces sustaining the target–ligand complex.

## 3. Discussion

In this work, Gracilioether A (GeA), a marine polyketide isolated from the sponge *Plakinastrella mamillaris*, already known for its antimalarial activity, was selected as an interesting probe for the identification of its interactomic profile to give more information on its biological potential. 

This experimental strategy was based on the combination of functional proteomics, biophysical techniques, and molecular docking. Limited proteolysis coupled to mass spectrometry techniques was applied to identify the potential target(s) and information on the region(s) of the interaction between GeA and its protein target.

USP5 (Ubiquitin carboxyl-terminal hydrolase 5) was found by the application of DARTS (Drug Affinity Responsive Target Stability) and was selected as the most promising partner of GeA among the list of its potential targets. USP5 belongs to the deubiquitinases, an enzyme class playing a crucial role in the control of various cellular pathological processes, such as inflammation and carcinogenesis. The following application of t-LiP-MS (targeted-Limited Proteolysis-Mass Spectrometry) provided information on the USP5 peptide region of interaction with GeA, some of which is located close to the ZnF-UBD (Zinc Finger Ubiquitin-Binding Domain). 

Surface Plasmon Resonance confirmed the direct interaction between GeA and USP5, measuring a K_D_ in the high micromolar range, while the identification of the possible binding mode and binding sites through molecular modelling, in accordance with the previous results (e.g., DARTS, t-LiP-MRM-MS and SPR), indicated ZnF-UBD as a primary binding site for GeA. All this evidence offer a comprehensive rationalization of the biological potential of GeA and opening the way for further steps of structure-based drug design.

## 4. Materials and Methods

### 4.1. Gracilioether A Extraction

*Plakinastrella mamillaris* Kirkpatrick, 1900 (order Homosclerophorida, family Plakiidae) was collected at a depth of 22 m from the Fiji Islands in May 2007. Taxonomic identification was performed by Dr John Hooper, Queensland Museum, Brisbane, Australia, where a voucher specimen was deposited under the accessing number G322695. The lyophilized material (171 g) was extracted with methanol (3 × 1.5 L) at room temperature, and the crude methanol extract (40 g) was subjected to a modified Kupchan’s partitioning procedure to obtain three extracts [4]. The CHCl_3_ extract (1.0 g) was chromatographed via MPLC using silica gel and a solvent gradient system from CH_2_Cl_2_ to CH_2_Cl_2_:MeOH 1:1. Fractions eluted with CH_2_Cl_2_:MeOH 99:1 were further purified via HPLC on a Nucleodur 100-5 C18 (5 μm; 10 mm i.d. × 250 mm) with 70% MeOH:H_2_O as eluent (flow rate 3.5 mL/min) to give 16.0 mg of Gracilioether A (4) (t_R_ = 26 min).

### 4.2. Cell Culture

HeLa cells were first grown in Dulbecco’s modified Eagle medium with 10% (*v*/*v*) fetal bovine serum albumin, 100 U/mL penicillin and 100 mg/mL streptomycin (Sigma–Aldrich, St. Louis, MO, USA) at 37 °C in 5% CO_2_ atmosphere and then collected via centrifugation (1000× *g*, 5 min). 

### 4.3. DARTS Experiment

The cells were then suspended in PBS supplemented with 0.1% *v*/*v* Igepal and protease inhibitor cocktail and mechanically lysed using a Dounce homogenizer at 4 °C. The resulting suspension was centrifuged (10,000× *g* for 5 min at 4 °C), the supernatant was collected for further analysis, and the protein concentration was determined using the Bradford assay (BioRad Laboratories, Hercules, CA, USA). The lysate was diluted to 3 μg/μL and divided into four equal aliquots; three of those were treated with different concentrations of GeA resuspended in DMSO, given its poor solubility in water. The fourth aliquot served as a control and was treated with an equal volume of DMSO. After an hour of incubation under continuous shaking, the limited proteolysis was carried out, treating the samples with different amounts of subtilisin for 30 min at 25 °C under continuous shaking. Protease was then quenched by adding PMSF (Sigma-Aldrich, St. Louis, MO, USA) to each sample. 

In this study, 20 µg of each sample were then boiled in Laemmli buffer (60 mM Tris–HCl pH 6.8, 2% SDS, 0.001% bromophenol blue, 1% glycerol, and 2% ß-mercaptoethanol) and loaded on a 4–12% Bis–Tris Criterion™ XT Precast Gel (Bio-Rad Laboratories S.r.l.). After electrophoretic separation performed using BioRad equipment (BioRad Laboratories), the proteins were stained through the use of Coomassie solution and gel bands relative to 1:500 *w*/*w* subtilisin ratio, resulting in the best proteolysis condition; they were cut out from the gels and submitted to an in situ tryptic digestion protocol [23]. Briefly, protein sulphide bonds were reduced with 6.5 mM 1,4-dithiothreitol (DTT), and thiols were then carboxyamidomethylated, adding 54 mM iodoacetamide (IAA). After hydration/dehydration cycles, 12 ng/µL trypsin/LysC solution (Promega, Madison, WI, USA) was added on ice for 1 h. After the addition of ammonium bicarbonate (30 µL, 50 mM, pH 8.5), the bands were incubated overnight at 37 °C to allow protein digestion. After this incubation, the peptides were extracted from the slices using 100% CH_3_CN (Deltek Srl, Pozzuoli, Italy) twice, and the peptide samples were dried under vacuum and dissolved in formic acid (FA, 10%) before MS analysis. Tryptic peptides were extracted from each gel slice, and the obtained peptide mixtures were dried under vacuum and dissolved in formic acid (FA, 10%) for LC-MS-MS analysis. 

A nano-flow RP-UPLC MS/MS was used to inject 1 µL of each sample, which was then analyzed with the Orbitrap Q-Exactive Classic Mass Spectrometer (ThermoFisher Scientific, Bremen, Germany) coupled to an UltiMate 3000 Ultra-High Pressure Liquid Chromatography (UPLC) system (ThermoFisher Scientific, Bremen, Germany) equipped with an EASY-Spray PepMAPTM RSLC C18 column (3 μm, 100 Å, 75 μm × 50 cm, ThermoFisher Scientific, Bremen, Germany). The gradient ranged from 1 min at 3% B up to 28% of B in 40 min (A: 95% H_2_O, 5% CH_3_CN, 0.1% AcOH; B: 95% CH_3_CN, 5% H_2_O, 0.1% AcOH), then from 28 to 49 min isocratic at 70% of B and at 49 min back to 3% of B for another 10 min before the starting of next run. MS analysis was operated in the data-dependent acquisition mode. The mass spectrometry proteomics data have been deposited to the ProteomeXchange Consortium via the PRIDE [24] partner repository with the data set identifier PXD048098.

Data analysis was carried out with Proteome Discoverer (Proteome Discoverer^TM^ Software, version 2.4, ThermoFisher Scientific, Bremen, Germany), which used in silico SwissProt data for protein identification and was used to perform a relative quantitative analysis comparing the protein abundance in each sample treated with GeA to that of the untreated sample.

Proteome Discoverer was used using the SwissProt database and the following parameters: maximum of two missed cleavages, trypsin digestion, and carbamidomethyl (C) as the fixed modification; oxidization (M) and protein N-terminal acetylation as variable modifications and using Sequest HT with multi-peptide search and percolator validation. 

Experiments were carried out in triplicate.

The filter criteria for protein selection were as follows: proteins with abundance ratio “lysate/ctrl”, as positive (lysate: sample with no GeA and undigested = maximum level of protein abundance) and negative controls (ctrl: sample with no GeA but digested = minimum level of protein abundance), <2 have been excluded. The list of remaining proteins is limited, considering only those with increasing abundances of GeA concentrations.

### 4.4. Western Blotting

Samples of one DARTS experiment were used for data validation through Western Blotting. In this study, 7 μL of each sample was treated with Laemmli buffer and heated at 95 °C for 5 min for the electrophoresis separation in a 10% SDS-PAGE gel. The proteins were then blotted on a nitrocellulose membrane, which was then dipped in 5% non-fat dried milk for 1 h at room temperature under continuous shaking and then incubated overnight at 4 °C with agitation with primary mouse antibodies against the protein USP5 (1:1000 *v*/*v*, Santa Cruz Biotechnology, Dallas, TX, USA). The membrane was then washed three times with TBS-t (31 mM Tris pH 8, 170 mM NaCl, 3.35 mM KCl, 0.05% Tween 20) to remove the excess antibody and re-incubated with a mouse peroxidase-conjugated secondary antibody (1:2500 *v*/*v*, Thermo-Scientific) at room temperature for 1 h under shaking. The signal was developed thanks to an enhancer solution combined with a peroxide solution (GeneSpin, Milano, Italy) and the signal was detected with LAS 4000 (GE Healthcare Waukesha, WI, USA). The procedure was repeated to incubate the membrane with the primary mouse anti-Glyceraldehyde 3-Phosphate Dehydrogenase antibody (GAPDH, 1:2500 *v*/*v*, Santa Cruz Biotechnology) to avoid errors due to gel loading.

### 4.5. Surface Plasmon Resonance

SPR is a powerful technique used to study biomolecular interactions in real time. The experiments were performed using a Biacore T200 instrument equipped with a research-grade CM5 sensor chip (Cytiva, Marlborough, MA, USA). Recombinant human Ubiquitin-specific proteinase 5 protein (USP5) obtained from Aviva Systems Biology (San Diego, CA, USA) was immobilized on the sensor chip surface using the standard amine-coupling protocol [25]. Protein immobilization was carried out at a concentration of 0.15 μg μL^−1^ in 10 mM CH_3_COONa, 25 mM, pH 4.5, with a contact time of 500 s at a flow rate of 10 μL min^−1^, which obtained an immobilization density of 8–9 kRU. Two surfaces were prepared for the experiments: one with immobilized USP5 protein and one unmodified reference surface. GeA, the compound of interest, was dissolved in 100% DMSO to obtain 10 mM solutions and further diluted to 1:20 (*v*/*v*) in PBS-P (PBS-P buffer: 0.2 M phosphate buffer, 27 mM KCl, 1.37 M NaCl, 0.5% surfactant P20) to a final DMSO concentration of 5.0%. The compound was injected in concentration series (1:2 dilution, 10 different concentrations) and prepared in 96-well plates, ranging from 0 to 100 μM. SPR experiments were conducted at 25 °C, using a flow rate of 20 μL min^−1^. The association phase was monitored for 180 s, followed by 300 s of dissociation [26]. The K_D_ value, which represents the equilibrium dissociation constant and indicates the strength of the interaction between the immobilized USP5 protein and the injected compounds, was determined using the Biaevaluation software performing a global fitting of the double-referenced association and dissociation data to a 1:1 interaction model.

### 4.6. t-LiP-MRM-MS: Methods Fine-Tuning

The Human build of the proteomic data repository Peptide Atlas was used to obtain information about the USP5 (UniProt Accession: P45974) tryptic peptides, which were then researched in the SRM Atlas to find their fragment ions. Thus, the three best transitions for each peptide were chosen to build MRM methods that were subsequentially tested on a tryptic-digested HeLa cell lysate obtained as described below.

The proteins were extracted as previously described and then denatured with urea 8 M/50 mM AmBic to a final concentration of 4 M urea. Disulfide bonds were reduced using 10 mM 1,4-dithiothreitol (DTT) for 1 h at 25 °C, 500 rpm (Thermomixer, Biosan, Riga, Latvia) and the resulting thiols were alkylated with 20 mM iodoacetamide (IAA) for 30 min, 25 °C, 500 rpm in the dark. The excess IAA was quenched with 10 mM DTT for 10 min at 25 °C, 500 rpm and then 50 mM AmBIc was used to dilute urea from 4 M to 1 M. Trypsin/Lys C mix (Promega, Madison, WI, USA) was added to the sample (enzyme-to-protein ratio of 1:100 *w*/*w*) overnight at 37 °C under shaking. After enzyme inactivation (lowering pH to 3 with formic acid), the peptides were dried under vacuum and resuspended in 1 mL 5% FA and desalted with Sep-Pak Vac C18 1 cc (50 mg) cartridges (Waters, Milford, MA, USA). The peptides were dried again under vacuum and re-dissolved in 10% FA for MRM analysis, which was performed in a positive MRM scanning mode on a 6500 Q-TRAP (AB Sciex, Toronto, ON, Canada) equipped with a Shimadzu LC-20A system. Chromatographic separation was performed on a Kinetex Aeris 3.6 µm Peptide XB C18 (50 × 2.1 mm) column using a gradient at 300 µL/min from 5% to 95% of B in 12 min (A: 0.1% Formic Acid in H_2_O: CH_3_CN (95:5), B: 0.1% Formic Acid in CH_3_CN: H_2_O (95:5).

### 4.7. Interactions Study of the Complex GeA/USP5: t-LiP-MRM-MS

HeLa cell lysate, obtained as previously described, was incubated with GeA at 100 μM concentration and with DMSO for 1 h at room temperature under shaking (Mini-Rotator, Biosan) and then treated with subtilisin (enzyme to proteins ratio of 1:1500 and 1:500) for 30 min at 25 °C under continuous shaking (500 rpm, Thermomixer, Biosan), leaving an undigested sample (with no GeA) as a control. Once the enzyme was quenched, the samples were first submitted to denaturant conditions with urea 4 M and then to the in-solution digestion protocol, as previously described. Thus, MRM-MS analysis was performed in triplicate with USP5 MRM methods already optimized. Analyst Software (AB Sciex) was used to measure the areas of each tryptic peptide peak. The experiments were carried out in triplicate.

### 4.8. Interactions of the Complex GeA/USP5: Computational Studies

The 3D structure of USP5 zinc finger ubiquitin binding domain co-crystallized with 3-(5-phenyl-1,3,4-oxadiazol-2-yl) propanoate (HHY) (PDB code: 6DXT [18]) was downloaded from the Protein Data Bank and was prepared using the Schrödinger Protein Preparation Wizard workflow [27,28] (Schrödinger Suite). Specifically, water molecules and co-complexed compounds were removed, cap termini were included, all hydrogen atoms were added, and bond orders were assigned. Eventually, the prepared .pdb files were converted into the final .mae files. The grid accounted for the subsequent molecular docking calculations was generated using the co-crystallized molecule as a reference (See Figure S3). The final coordinates of the grid center were 23.05 (x), 42.94 (y), and −16.43 (z), and the gride featured inner and outer box dimensions of 10 × 10 × 10 and 23.10 × 23.10 × 23.10, respectively. 

The 2D structure of GeA was drawn with the 2D sketcher tool (Maestro, Schrödinger Suite [29] and was then processed using LigPrep software [30] (Schrödinger Suite 2021-1). All the possible tautomers and protonation states at pH = 7.4 (1.0) were generated, and the obtained structures were minimized using the OPLS 2005 force field. 

Molecular docking experiments were performed using Glide software [20,21,22,31] (Schrödinger Suite) in the Extra Precision (XP) mode. In detail, 10,000 poses were kept in the starting phase of docking and were evaluated to select 800 conformations to enter the minimization step with an energetic cutoff of 0.15 kcal/mol. After that, 20 maximum number of poses for the subsequent analysis were saved for visual inspections. 

## 5. Conclusions

In conclusion, the interaction profile of Gracilioether A (GeA) has been investigated, highlighting USP5 as an intriguing target for this molecule. The enzyme in question belongs to the deubiquitinase family and is involved in inflammation and various cancer processes, where it stabilizes several oncogenes. This study has confirmed the interaction of this molecule with this interesting enzyme, paving the way for further investigation of the biological potential of GeA in these pathological contexts. Indeed, future SAR studies may elucidate the structural moieties responsible for the affinity between the counterparts, which is useful for the rational design of new GeA analogs that are able to link or interfere with USP5.

## Data Availability

All data are contained within the article, SM and ProteomeXchange Consortium via the PRIDE (data set identifier PXD048098).

## Supplementary Materials

The following supporting information can be downloaded at: https://www.mdpi.com/article/10.3390/md22010041/s1, Figure S1: List of transitions of USP5 tryptic peptides selected for the analysis of the LiP experiment; Figure S2: USP5 peptides identified with the LiP experiment involved in binding with GeA; Figure S3: Binding mode of HHY (in violet) superimposed to its pose (in green) in the crystal structure of USP5 (PDB code: 6DXT); Figure S4: Uncropped image displaying the gel related to one of the DARTS experiments; Figure S5: Uncropped image of the Western blot confirmation of DARTS experiments; Figure S6: Darts data for USP5; Figure S7: Peak area of peptide transition analyzed in the t-LiP experiments; Figure S8: Plot of the RU values obtained through SPR analysis in function of GeA concentrations [16,19,20,21,22].
