# Supplementary material for: Chemoproteomics Reveals USP5 (Ubiquitin Carboxyl-Terminal Hydrolase 5) as Promising Target of the Marine Polyketide Gracilioether A
Source: Mar Drugs. 2024 Jan 11;22(1):41. doi: 10.3390/md22010041 (PMC10817451; doi:10.3390/md22010041)
Supplement: Supplementary file 1 [file marinedrugs-22-00041-s001.zip › marinedrugs-2784306-Supplementary-Supporting GeA_rev_22122023_final.pdf]

Chemoproteomics reveals USP5 (ubiquitin carboxyl-terminal hydrolase 5) as promising target of the marine polyketide Gracilioether A

Alessandra Capuano [1,2], Gilda D'Urso [1], Michela Aliberti[1,2], Dafne Ruggiero [1], Stefania Terracciano[1], Carmen Festa[3], Alessandra Tosco [1,], Maria Giovanna Chini [4], Gianluigi Lauro [1], Giuseppe Bifulco [1] and Agostino Casapullo [1]*

Selection of USP5 tryptic peptides for t-LiP experiments

The choice was made after testing various transitions for each peptide on a simple triptic digest of HeLa cell. The peptide listed in the table provided a better response in terms of peak signal intensity and signal-to-noise ratio, on the employed spectrometer, The selected transitions are related to the peptides highlighted in yellow. These peptides constitute 48.8% of the total sequence of the target protein. The selection of transitions ensures a more than satisfactory coverage of the USP5 sequence.

Q1_mz	Q3_mz	Peptide
788.913	693.3168	V-[147-162]-R
782.3992	276.1559	L-[99-113]-K
722.3705	899.4838	S-[779-793]-K
936.5115	203.0854	M-[1-17]-R
556.3095	599.3517	D-[139-144]-R
707.9194	976.5104	I-[122-133]-R
770.9247	1088.6356	F-[562-574]-K
589.776	875.4375	S-[453-462]-R
669.3304	776.3731	D-[846-855]-R
407.2149	572.2792	Q-[185-191]-R
533.2724	780.3892	W-[829-836]-K
497.7406	277.1188	F-[114-121]-K
591.2828	697.3045	E-[501-509]-K
671.3000	259.0930	E-[81-93]-R
930.9678	244.0933	E-[206-221]-R
464.7684	203.0854	M-[517-524]-R
489.2693	231.0981	E-[239-247]-K
658.8157	258.1090	Q-[164-174]-R
703.3486	895.4525	I-[361-379]-K
608.2685	756.8329	Y-[223-238]-R
663.0071	731.4052	L-[587-603]-R
706.6866	257.125	Q-[433-449]-R
952.8485	758.4383	I-[304-329]-R
892.7469	676.3266	A-[755-778]-R
339.1801	260.1069	V-[837-845]-K
348.8340	425.7069	G-[424-432]-R
571.7861	557.7806	L-[380-401]-R
959.4948	1059.5549	V-[483-499]-K
740.8427	463.1863	T-[292-303]-R
429.2211	372.6791	I-[192-198]-K
492.7596	713.347	E-[407-415]-R

MAELSEEALLSVLPTIRVPKAGDRVHKDECAFSFDTPESEGGLYIC
MNTFLGFGKQYVERHFNKTGQRVYLHLRRTRRPKEEDPATGTGD
PPRKKPTRLAIGVEGGFDLSEEKFELDEDVKIVILPDYLEIARDGLG
GLPDIVRDRVTSAVEALLSADSASRKQEVQAWDGEVRQVSKHA
FSLKQLDNPARIPPCGWKCSKCDMRENLWLNLTDGSILCGRRYF
DGSGGNNHAVEHYRETGYPLAVKLGTITPDGADVYSYDEDDMV
LDPSLAEHLSHFGIDMLKMQKTDKTMTELEIDMNQRIGEWELI
QESGVPLKPLFGPGYTGIRNLGNSCYLNSVVQVLFSIPDFQRKYV
DKLEKIFQNAPTDPTQDFSTQVAKLGHGLLSGEYSKPVPESGDGE
RVPEQKEVQDGIAPRMFKALIGKGHPEFSTNRQQDAQEFFLHLI
NMVERNCRSSENPNEVFRFLVEEKIKCLATEKVKYTQRVDYIMQL
PVPMDAALNKEELLEYEEKKRQAEEEKMALPELVRAQVPFSSCLE
AYGAPEQVDDFWSTALQAKSVAVKTTRFASFPDYLVIQIKKFTFGL
DWVPKKLDVSIEMPEELDISQLRGTGLQPGEEELPDIAPPLVTPD
EPKGSLGFYGNEDEDSFCSPHFSSPTSPMLDESVIIQLVEMGFPM
DACRKAVYYTGNSGAEAAMNWVMSHMDDPDFANPLILPGSS
GPGSTSAAADPPPEDCVTTIVSMGFSRDQALKALRATNNSLERA
VDWIFSHIDDLDAEAAMDISEGRSAADSISESVPVGPKVRDGPG
KYQLFAFISHMGTSTMCGHYVCHIKKEGRWVIYNDQKVCASEKP
PKDLGYIYFYQRVAS

Figure S1: List of transitions of USP5 tryptic peptides selected for the analysis of the t-LiP experiment.

Identification of the USP5 peptides involved in the binding with GeA

In Figure S2 the Fold change (Fc) represents the abundance of the peptide in the sample treated with GeA compared to the untreated sample. The presence of the molecule has exerted a protective effect in that region, preventing subtilisin from digesting the peptide. Therefore, the peptide abundance is higher in the treated sample, and thus, a satisfactory Fold change is represented by a positive ratio. Abundance is measured by the area under the chromatographic peak of the peptide. In the calculation of Fold change, the abundance of the peptide is compared between the positive control and the negative control. The positive control represents the sample untreated with the molecule and undigested with subtilisin (being undigested, it serves as the reference for the maximum quantity of peptide in the cell). The negative control is the sample untreated with the molecule but digested with subtilisin (without the molecule to protect, subtilisin exerts its maximum action, resulting in the lowest concentration of peptide among all analyzed samples). This ratio must always be positive and high, indicating that digestion has caused sufficient disturbance in the system to assess variations in Fold change in the treated samples.

			Treated sample		lysate	
Q1_mz	Q3_mz	peptide	Fc	p-value	Fc	p-value
788.913	693.3168	V-[147-162]-R	3.76	0.04	15.57	0.021
703.3486	895.4525	I-[361-379]-K	2.16	0.021	8.15	0.012
571.7861	557.7806	L-[380-401]-R	2.17	0.001	2.69	0.046
722.3705	899.4838	S-[779-793]-K	5.32	0.007	7.6	0.012
492.7596	713.347	E-[407-415]-R	1.85	0.038	3.74	0.029

Figure S2: USP5 peptides identified with the LiP experiment involved in binding with GeA.

Re-docking of the compound HHY originally co-crystallized in the protein structure of USP5

To set molecular docking parameters, we used the compound HHY, originally co-crystallized in the protein structure of USP5 (PDB code: 6DXT[1]) as reference. In particular, we used Glide software[2-4] (Schrödinger Suite) in the Extra Precision (XP) mode to assess whether the binding mode of the compound HHY could be correctly reproduced. The results of these calculations showed that the generated molecular docking pose reproduced the same binding mode observed for the co-crystallized form of this ligand (Figure S3).

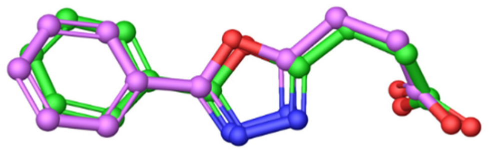

Figure S3. Binding mode of the HHY (in violet) superimposed to its pose (in green) in the crystal structure of USP5 (PDB code: 6DXT).

Gel-stained analysis of the DARTS experiment

The gel shows the loading of DARTS samples digested with various subtilisin ratios. However, the observation of the gel allowed us to appreciate increasing intensities in the 1:500 subtilisin ratios, therefore, this defect was considered for spectrometric analysis.

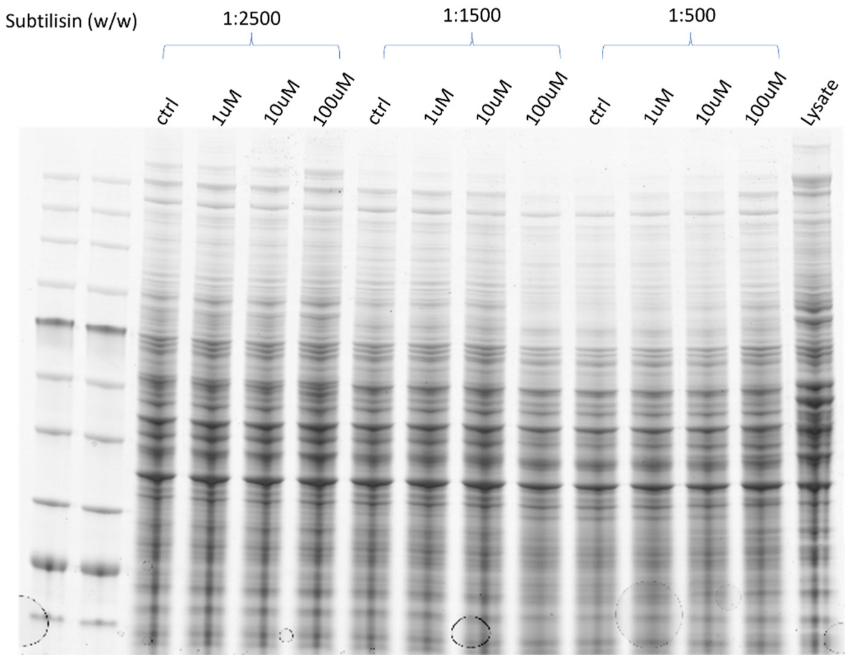

Figure S4: Uncropped image displaying the gel related to one of the DARTS experiments.

Western Blot analysis of the DARTS experiment

Figure S5: on the left side, the signal related to USP5. The same membrane was incubated with anti-GAPDH antibody (on the right side)[5]. Once again, samples treated with two subtilisin concentrations (enzyme to protein ratio 1:1500 and 1:500 (w/w), respectively) were loaded onto the gel and then transferred to the membrane and again, the 1:500 dilution revealed the best signal, indicating a residual amount of protein after digestion increasing in accordance with the concentration of the added molecule to the samples.

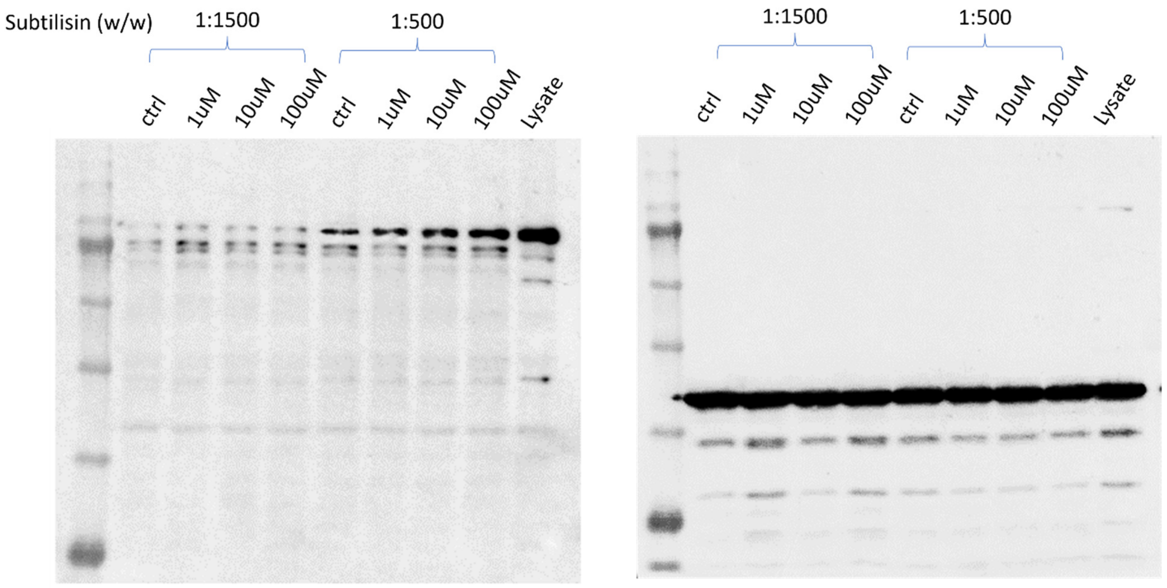

Figure S5: Uncropped image of the western blot confirmation of DARTS experiments.

Results of DARTS experiment

Protection of GeA on USP5 against subtilisin in the three DARTS experiments. The values indicate the ratio between the peptide abundance of samples treated with the three concentrations of the molecule compared to that of the control sample (digested without the molecule). The data show that in all three experiments, the molecule exerted a concentration-dependent protective effect, as inferred from the ratios that increase according to the concentration of GeA. Lys/ctrl indicates the ratio between positive control (no GeA, no subtilisin) and negative control (digested without the molecule). See also PD files attached as .XLS.

	Ubiquitin carboxyl-terminal hydrolase 5			
	1uM/ctrl	10uM/ctrl	100uM/ctrl	Lys/ctrl
Replicate A	6,457	6,537	11,423	22,634
Replicate B	1,61	2,195	3,497	31,495
Replicate C	1,374	3,739	8,385	16,375

Figure S6: DARTS data for USP5 in three experiments.

Results of t-LiP experiment

List of all the USP5 peptides analyzed in the t-LiP experiment with their peak area in different conditions (Ctrl: Sample digested without molecule; 100µM: sample treated with GeA100µM; Lysate: sample undigested without GeA). Among this list, five peptides were found to be involved in the binding according to Fold change values and p-values (see also Fig.S2)

		Peak Area					
		ctrl		100µM		Lysate	
Q1m/z	Q3m/z	Mean	Ds	Mean	Ds	Mean	Ds
722,3705	899,4838	1,35E+04	7,85E+03	7,20E+04	2,08E+03	1,03E+05	1,49E+04
788,913	693,3168	3,06E+03	1,61E+03	1,15E+04	7,07E+02	4,76E+04	1,29E+04
571,7861	557,7806	7,79E+05	7,21E+04	1,69E+06	1,19E+05	2,10E+06	2,79E+05
492,7596	713,347	2,94E+04	9,79E+03	5,45E+04	6,39E+03	1,10E+05	3,87E+04
703,3486	895,4525	1,39E+04	8,27E+03	2,99E+04	5,79E+03	1,13E+05	1,25E+04
407,2149	572,2792	4,88E+04	2,19E+04	1,04E+07	1,74E+07	2,01E+05	3,16E+04
497,7406	277,1188	1,68E+06	4,65E+05	2,08E+06	2,04E+05	2,24E+06	2,00E+05
533,2724	780,3892	5,04E+03	3,52E+03	1,87E+04	1,00E+03	3,46E+04	2,19E+03
556,3095	599,3517	2,10E+04	6,44E+03	5,05E+04	3,31E+04	5,64E+04	2,69E+04
589,776	875,4375	1,16E+04	1,15E+04	1,86E+04	9,26E+03	2,12E+04	8,65E+03
591,2828	697,3045	2,45E+04	7,35E+03	9,00E+04	5,96E+03	5,62E+05	2,86E+04
669,3304	776,3731	2,60E+03	6,17E+02	3,90E+04	2,90E+03	5,68E+04	5,94E+03
671,3000	259,0930	1,26E+05	6,40E+04	4,98E+05	2,17E+04	4,66E+05	4,36E+04
707,9194	976,5104	1,77E+04	1,19E+04	9,96E+04	1,63E+04	7,21E+04	5,39E+04
770,9247	1088,636	1,63E+03	1,43E+03	3,87E+03	3,65E+03	2,91E+04	7,13E+03
782,3992	276,1559	6,84E+05	1,69E+02	2,88E+06	6,93E+02	2,10E+06	5,57E+03
930,9678	244,0933	9,18E+03	1,61E+03	2,58E+04	4,44E+03	2,23E+04	1,29E+04
936,5115	203,0854	5,45E+03	4,09E+03	2,96E+04	2,33E+03	3,42E+04	1,71E+03
339,1801	260,1069	6,44E+05	2,97E+03	8,83E+05	7,53E+03	6,95E+05	5,52E+03
348,8340	425,7069	2,24E+05	2,75E+05	3,06E+05	1,21E+05	2,87E+05	9,47E+04
429,2211	372,6791	6,20E+05	8,82E+04	8,83E+05	3,63E+04	6,05E+05	7,00E+04
464,7684	203,0854	9,47E+04	2,70E+05	1,26E+05	7,17E+04	9,51E+04	1,54E+05
489,2693	231,0981	4,43E+05	4,03E+04	6,51E+05	2,65E+03	1,11E+06	1,68E+04
492,7596	713,347	1,80E+05	2,00E+05	2,42E+05	5,44E+04	2,86E+05	1,50E+05
608,2685	756,8329	1,69E+05	7,67E+05	1,59E+05	8,08E+04	2,10E+05	2,20E+05
658,8157	258,1090	1,16E+05	2,67E+04	1,18E+05	2,01E+03	1,52E+05	4,76E+04
663,0071	731,4052	1,03E+04	7,29E+04	1,73E+04	5,29E+03	3,98E+05	1,26E+04
706,6866	257,125	4,00E+05	4,85E+04	3,09E+05	7,02E+03	4,78E+05	1,60E+04
740,8427	463,1863	5,04E+04	5,75E+03	8,42E+04	1,89E+03	6,32E+04	5,97E+04
892,7469	676,3266	1,10E+04	8,53E+02	1,75E+04	5,63E+02	5,10E+04	9,90E+03
952,8485	758,4383	1,65E+04	3,59E+05	2,71E+04	6,61E+04	3,79E+04	6,86E+04
959,4948	1059,555	1,37E+04	2,36E+04	2,37E+04	7,19E+03	3,13E+04	2,40E+04

Figure S7: Peak area of tryptic peptides analyzed through t-LiP.

Results of SPR experiment

An additional plot has been obtained for SPR experiments, fitting RU vs GeA concentration.

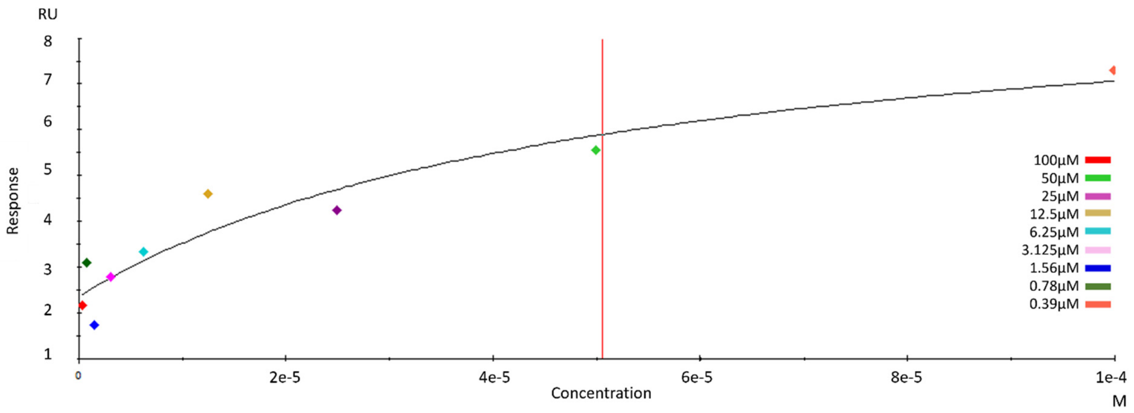

Figure S8: Graph relative to SPR data. The equilibrium responses were plotted as a function of GeA concentration.

Reference

1. Mann, M.K.; Franzoni, I.; de Freitas, R.F.; Tempel, W.; Houliston, S.; Smith, L.; Vedadi, M.; Arrowsmith, C.H.; Harding, R.J.; Schapira, M. Discovery of Small Molecule Antagonists of the USP5 Zinc Finger Ubiquitin-Binding Domain. *J Med Chem* **2019**, *62*, 10144-10155, doi:10.1021/acs.jmedchem.9b00988.
2. Halgren, T.A.M., R. B.; Friesner, R. A.; Beard, H. S.; Frye, L. L.; Pollard, W. T.; Banks, J. L. Glide: A New Approach for Rapid, Accurate Docking and Scoring. 2. Enrichment Factors in Database Screening. *J Med Chem* **2004**, *47 (7)*, 1750–1759.
3. Friesner, R.A.; Banks, J.L.; Murphy, R.B.; Halgren, T.A.; Klicic, J.J.; Mainz, D.T.; Repasky, M.P.; Knoll, E.H.; Shelley, M.; Perry, J.K.; et al. Glide: A new approach for rapid, accurate docking and scoring. 1. Method and assessment of docking accuracy. *J Med Chem* **2004**, *47*, 1739-1749, doi:10.1021/jm0306430.
4. Friesner, R.A.; Murphy, R.B.; Repasky, M.P.; Frye, L.L.; Greenwood, J.R.; Halgren, T.A.; Sanschagrin, P.C.; Mainz, D.T. Extra precision glide: Docking and scoring incorporating a model of hydrophobic enclosure for protein-ligand complexes. *J Med Chem* **2006**, *49*, 6177-6196, doi:10.1021/jm051256o.
5. Pai, M.Y.; Lomenick, B.; Hwang, H.; Schiestl, R.; McBride, W.; Loo, J.A.; Huang, J. Drug affinity responsive target stability (DARTS) for small-molecule target identification. *Methods Mol Biol* **2015**, *1263*, 287-298, doi:10.1007/978-1-4939-2269-7_22.
